# Chlorophyllase (PsCLH1) and light-harvesting chlorophyll *a*/*b* binding protein 1 (PsLhcb1) and PsLhcb5 maintain petal greenness in *Paeonia suffruticosa* ‘Lv Mu Yin Yu’

**DOI:** 10.1016/j.jare.2024.09.003

**Published:** 2024-09-03

**Authors:** Qing Hao, Tongtong Li, Gaojie Lu, Shuo Wang, Zhen Li, Cancan Gu, Fan Kong, Qingyan Shu, Yang Li

**Affiliations:** a College of Landscape Architecture and Forestry, Qingdao Agricultural University, Qingdao 266109, China; b State Key Laboratory of Plant Diversity and Specialty Crops, Institute of Botany, Chinese Academy of Sciences, Beijing 100093, China; c China National Botanical Garden, Beijing 100093, China; d University of Chinese Academy of Sciences, Beijing 100049, China; e College of Agricultural Science and Engineering, Liaocheng University, Liaocheng 252000, China

**Keywords:** Tree peony, Chlorophyll degradation, Petal degreening, Chlorophyllase, Light-harvesting chlorophyll *a*/*b* binding protein

## Abstract

**Introduction:**

Green flowers are not an adaptive trait in natural plants due to the challenge for pollinators to discriminate from leaves, but they are valuable in horticulture. The molecular mechanisms of green petals remain unclear. Tree peony (*Paeonia suffruticosa*) is a globally cultivated ornamental plant and considered the ‘King of Flowers’ in China. The *P*. *suffruticosa* ‘Lv Mu Yin Yu (LMYY)’ cultivar with green petals could be utilized as a representative model for understanding petal-specific chlorophyll (Chl) accumulation and color formation.

**Objectives:**

Identify the key genes related to Chl metabolism and understand the molecular mechanism of petal color changes.

**Methods:**

The petal color parameter was analyzed at five developmental stages using a Chroma Spectrophotometer, and Chl and anthocyanin accumulation patterns were examined. Based on comparative transcriptomes, differentially expressed genes (DEGs) were identified, among which three were functionally characterized through overexpression in tobacco plants or silencing in ‘LMYY’ petals.

**Results:**

During flower development and blooming, flower color changed from green to pale pink, consistent with the Chl and anthocyanin levels. The level of Chl demonstrated a similar pattern with petal epidermal cell striation density*.* The DEGs responsible for Chl and anthocyanin metabolism were characterized through a comparative transcriptome analysis of flower petals over three critical developmental stages. The key chlorophyllase (PsCLH1) and light-harvesting chlorophyll *a*/*b* binding protein 1 (PsLhcb1) and PsLhcb5 influenced the Chl accumulation and the greenness of ‘LMYY’ petals.

**Conclusion:**

PsCLH1, PsLhcb1, and PsLhcb5 were critical in accumulating the Chl and maintaining the petal greenness. Flower color changes from green to pale pink were regulated by the homeostasis of Chl degradation and anthocyanin biosynthesis. This study offers insights into underlying molecular mechanisms in the green petal and a strategy for germplasm innovation.

## Highlights

• The petal color changed from green to pink was consistent with the content of chlorophyll and anthocyanin.
• The chlorophyll content showed a similar pattern with petal epidermal cell striation density.
• PsCLH1 regulated chlorophyll accumulation and promoted the degreening of petals.
• PsLhcb1 and PsLhcb5 regulated chlorophyll accumulation and repressed the degreening of petals.
• The petal color changes through a balance of chlorophyll degradation and anthocyanin biosynthesis.

## Graphical abstract

**Figure:**
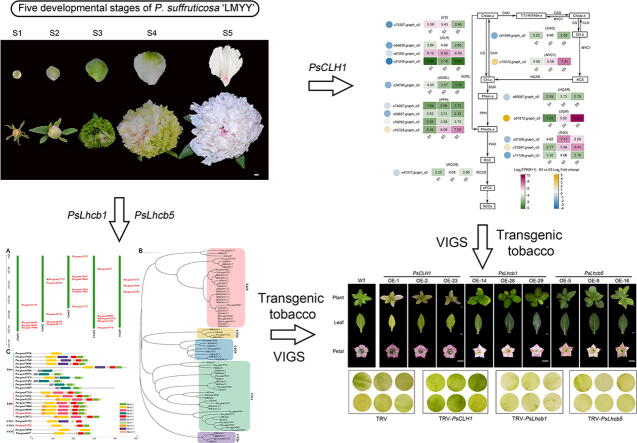

## Introduction

Flower color is an adaptive phenotypic trait throughout the evolution of plant’ species. In the horticultural field, it represents a quality trait in ornamental plants and is pivotal in improving commercial value as well as yield [1]. Due to their distinctive features and scarcity, green flowers are very popular, with great commercial value. These plants have been the subject of considerable scientific interest in plant evolution, adaptation, and breeding since this is not an optimal trait for natural plants due to the interaction between pollinating insects and flowers. Growing green flowers has become a goal for breeders, such as chrysanthemums and carnations as cut flowers and garden decorations worldwide [2], [3], [4]. Molecular research into the regulation of the green color formation of flowers has only been conducted in a limited number of species, including *Lilium* ‘Tiny Padhye’ [5], *Dendrobium officinale* [6], and chrysanthemum [4]. The absence of identified molecular mechanisms limits the breeding of green flowers and fails to satisfy the public’s goals. The color of a flower is governed by the pigments accumulated in the petals and their respective concentrations. Green petals are formed due to chlorophyll (Chl) biosynthesis and accumulation. Petals are derived from leaves [7], and green petals may be photosynthetic. Consequently, genes associated with green petal formation are also linked to our understanding of Chl biosynthesis and metabolism, particularly for related gene expression differences compared to leaves. The investigation of petal greening and other typical coloring (e.g., pink) makes it an ideal model for paving the way to understanding the balance between Chl and other pigments. Consequently, studying the mechanism underlying the production of green flowers through examining the regulation of color formation is necessary, particularly in woody ornamental plants due to their long life span and desirable characteristics.

The tree peony (*Paeonia suffruticosa* Andrew) is a well-known global ornamental woody plant, widely cultivated as a garden plant, potted flower, and cut flower [8]. It has been cultivated in China as the ‘King of Flowers’ for over 2,000 years with various colors including pink, red, purple, and white, among which green-colored flowers are relatively rare [9]. Therefore, a few cultivars, e.g., ‘Lv Mu Yin Yu (LMYY)’, ‘Chun Liu,’ and ‘Dou Lv’, with green flowers have higher commercial value due to their beauty and rarity. The tree peony flowers were divided into five developmental stages designated S1 (tight flower bud stage), S2 (swollen flower bud stage), S3 (initiating blooming stage), S4 (cup-blooming stage), and S5 (the blooming stage), respectively [10]. However, these green-colored cultivars only have green buds and petals change from green to white or pink during blooming. The mechanism of maintaining green in tree peony flowers during blooming remains undefined. Petal pigments are divided into four categories: flavonoids, Chl, carotenoids, and betalains [11]. The balance between Chl degradation and anthocyanin biosynthesis governs plant coloration, such as fruits in *Litchi chinensis*, leaves in ornamental kale, and barks in willow [12], [13], [14]. A few related studies have been performed on flower petals due to the lack of typical model plant with transitioning from green to other colors [15]. The molecular mechanism of the balance must be investigated using more plant’ species.

Chl is essential for green-colored flowers in plants [16] and present in the early developmental stages of flowering plant petals and gradually degraded during blooming [6]; therefore, repressing the Chl degradation is a substantial challenge. The Chl metabolic pathway comprises three steps: 1) synthesis of Chl *a*; 2) interconversion of Chl *a* and *b* (Chl cycle); and 3) degradation of Chl *a* [17]. Chlorophyllase (CLH), as an esterase, can catalyze the cleavage of the hydrophobic thylakoid-anchoring phytol chain of Chl from the porphyrin ring, producing chlorophyllide (Chlide), including Chl *a*/*b* to Chlide *a*/*b*. CLH is considered a rate-limiting enzyme for Chl degradation [18]. Chl *a* is degraded to pheophytin a (Phein *a*) by the Mg-dechelatase STAY-GREEN (SGR), and Phein *a* is deacetylated to pheophorbide *a* (Pheide *a*) by pheophytinase (PPH). Chlide *a* is also directly dechelated to Pheide *a* by SGR-like (SGRL) enzymes. Finally, pheophorbide *a* oxygenase (PAO) opens the tetrapyrrole ring to produce a linear form for the downstream degradation pathway [19], [20]. The Chl degradation has been studied in the petals of some plants, including *Lilium* species [5] and *Petunia hybrida* [2]. Compared to Chl degradation, the light-harvesting Chl *a*/*b* binding (Lhc) proteins in plastids are important for maintaining greening, associated with photosystem (PS) I and II, named Lhca (or LHCI) and Lhcb (or LHCII), respectively [21]. They are also involved in development processes, such as seed germination and post-germination [22], and abiotic stress tolerance [23], [24], [25], [26]. However, the repression of Chl degradation in maintaining the green color of tree peony petals remains unclear.

In our investigation, we employed the representative cultivar *P. suffruticosa* ‘LMYY’ with green flowers as a research model to determine the molecular mechanism of petal degreening. The petal color parameter was first analyzed at five developmental stages, and then the Chl and anthocyanin accumulation patterns were investigated. Based on comparative transcriptomes, the key differentially expressed genes (DEGs) were functionally characterized *in vivo*.

## Materials and methods

### Plant materials

The cultivar *P. suffruticosa* ‘LMYY’ with green flowers was cultivated at the Qingdao Agricultural University (Qingdao, China) experimental farm, located at Lat. 36°19’4’’ N, 120°23’11’’ E, Alt. 37 m, with maximum temperature, minimum temperature, and average relative humidity of 33.74°C, −6.86°C, and 74.93% in 2022, respectively (https://www.weatherspark.com/). Five representative developmental floral stages were [10]: S1 (tight flower bud stage), S2 (swollen flower bud stage), S3 (initiating blooming stage), S4 (cup-blooming stage), and S5 (blooming stage). Flower samples were obtained and stored at −80°C for further analysis. *Nicotiana tabacum* cv. Nc89 was grown as described previously [27].

### Flower color parameter measurements

The International Commission on Illumination (CIE) recommended the use of two approximately uniform color spaces and color difference formulas (CIELAB space and CIELUV) in 1976 [28]. The CIELAB space (L*a*b*) has been widely employed to describe the color of fruits, leaves, and flowers. The value of L* ranged from black (0) to white (1 0 0), a* ranged from red (positive) to green (negative), and b* ranged from yellow (positive) to blue (negative). The CIEL*a*b* color system was utilized as previously described [29], using a Chroma Spectrophotometer CR-400 (Konica Minolta, Japan). Measurements were conducted on the middle section on the adaxial sides of petals from five individual flowers as replicates. The mean values were employed for analysis.

### Determining Chl and anthocyanin levels

Total Chl, Chl *a*, and Chl *b* levels were measured as previously described [30]. Specifically, petals underwent extraction in 95% ethanol for 48 h in the dark at 4°C. The extraction solution was then evaluated using a UV photometer at 645 and 663 nm. The Chl levels (μg/g FW) were computed as follows [V was the extraction solution volume (mL), A was the absorbance, *m* was the petals FW (g)]:

$$Total\;Chl\;\left(\mu g/g\right)=\frac{(20.29\times A_{663}+8.05\times A_{645})\times V}{m}$$

$$Chl\;a\;\left(\mu g/g\right)=\frac{(12.72\times A_{663}-2.59\times A_{645})\times V}{m}$$

$$Chl\;b\;\left(\mu g/g\right)=\frac{(22.88\times A_{663}-4.67\times A_{645})\times V}{m}$$

The total anthocyanin content was identified as previously described [10], petals were dipped in 0.1 mol/L HCl-CH_3_OH solution (1: 99, 1 mol/L HCl: 1 mol/L CH_3_OH, v/v) for 24 h at 4°C. The extraction solution was measured using a UV photometer at 530, 620, and 650 nm. The total anthocyanin content was determined as follows [V was the extraction solution volume (mL), A was the absorbance, *m* was the petals FW (g), ε, the molar extinction coefficient of anthocyanin, is 4.62 × 10^6^]:

$$A_{\lambda }=\left(A_{530}-A_{620}\right)-0.1\times \left(A_{650}-A_{620}\right)$$

$$Total\;anthocyanin\;\left(nmol/g\right)=\frac{A_{\lambda }}{\varepsilon }\times \frac{V}{m}\times 1000000$$

### Epidermal cells observation

The petals from each floral developmental stage were cut into small squares (approximately 5 mm^2^ in size) and fixed in FAA solution for 48 h, followed by dehydration using an ethanol gradient series of 50, 70, 80, 90, and 100%, for 5 min each. Absolute ethanol was finally replaced with isoamyl acetate, samples were dried in an Emitech K850 Critical Point Dryer, and coated with platinum using a JFC-1600 Ion Sputter before examining under a JEOL 7500F scanning electron microscope (Peabody, USA) [30].

### Transcriptome sequencing and DEG analysis

The petals at three critical developmental stages (S1, S3, and S5) were utilized for transcriptome sequencing, and data were obtained via sequencing by synthesis (SBS) technology at Biomarker Technologies (Beijing, China) using the Illumina HiSeq 2000 platform. After removing adapter sequences and low-quality reads, high-quality clean data was obtained for sequence assembly.

Ebseq was used for differential expression analysis to identify the DEGs between two samples as a comparison group [31]. The screening criteria were FDR (false discovery rate) < 0.001 and |fold change| ≥ 2. GO and KEGG pathway enrichment analyses of the DEGs were conducted using the topGO R package based on the Kolmogorov-Smirnov test and KOBAS software, respectively. Based on enrichment analysis, DEGs involved in Chl degradation and anthocyanin biosynthesis were screened further.

### Quantitative RT-PCR (qRT-PCR) analysis

The qRT-PCR analysis was performed as previously described [32] using SYBR qPCR Master Mix (Vazyme, China). *PsTubulin* (*PsTUB*) was utilized as an internal control. The primers are presented in Table S1.

### *PsCLH1, PsLhcb1*, and *PsLhcb5*-overexpressing tobacco plant generation

The coding sequences of *PsCLH1*, *PsLhcb1*, and *PsLhcb5* were cloned into pSuper1300 [33]. Hygromycin B-resistant plantlets were transferred to soil and cultivated in pots, and T_2_ transgenic lines were employed for further analysis. The primers are listed in Table S1.

### Virus-induced gene silencing (VIGS)

VIGS was conducted using previously described methods [34]. Gene-specific fragments of *PsCLH1* (379 bp), *PsLhcb1* (306 bp), and *PsLhcb5* (339 bp) were used to construct the pTRV-*PsCLH1*, pTRV-*PsLhcb1*, and pTRV-*PsLhcb5* vectors, which were transformed into *Agrobacterium tumefaciens* GV3101 strain. The transformed GV3101 cells were resuspended in infiltration buffer (10 mM MgCl_2_, 200 mM acetosyringone, and 10 mM 2-(N-Morpholino)-ethanesulfonic acid, pH 5.6), and a mixture of GV3101 cultures containing an equal volume of pTRV1 + pTRV2-*PsCLH*/*PsLhcb1*/*PsLhcb5*, respectively, or pTRV1 + pTRV2 as the negative control. The petals were infiltrated by vacuuming and cultured in an incubator at 8°C for 3 days in the dark and then placed at 23°C for 8 days under a 14-hour light/10-hour dark photoperiod. After the culturing period, the petals were used for qRT-PCR analysis and Chl content characterization. All primers used for VIGS are presented in Table S1.

### Identification of *Lhcs* in the *P. ostii* genome and gene structure analysis

The sequences of *Lhcs* from *P. suffruticosa* ‘LMYY’ transcriptome and *P*. *ostii* genome database (https://ftp.cngb.org/pub/CNSA/data5/CNP0003098/CNS0560369/CNA0050666/) were utilized to identify possible members using TBtools bidirectional BLAST alignment [35]. After the identification of conserved domains on NCBI CDD (https://www.ncbi.nlm.nih.gov/cdd/), *PoLhcs* were used for mapping chromosomal positions and structure analysis via TBtools.

### Phylogenetic analysis and conserved motif prediction of PoLhcs

Phylogenetic analysis was conducted as described previously [36]. The amino acid sequences of Lhcs in *A*. *thaliana* and *Malus domestica* were used as references and obtained from TAIR (https://www.arabidopsis.org) and the apple genome database (https://www.rosaceae.org/), respectively. The phylogenetic tree of 23 PoLhcs, 21 AtLhcs, and 27 MdLhcs was constructed using the Neighbor-Joining method based on a *p*-distance model within MEGA X software and iTOL (https://itol.embl.de/). The analysis included 71 amino acid sequences with pairwise deletion.

For motif analysis, the PoLhcs amino acid sequences were submitted to MEME (https://meme-suite.org/meme/tools/meme) with a maximum of 10 motifs and visualized by TBtools.

### Co-expression network and promoter analysis

Co-expressed genes and their function were analyzed by string database (https://www.string-db.org) and visualized using Cytoscape and OmicShare tools (https://www.omicshare.com). *Cis*-elements were queried from PlantCARE (bioinformatics.psb.ugent.be/webtools/plantcare/html) and visualized with TBtools.

### Statistical analysis

Statistical analysis was conducted using GraphPad Prism (version 10.0). Using the two-tailed Student’s *t* and one-way ANOVA multiple comparisons test to analyze experimental data, differences were considered significant at *P* < 0.05, 0.01, or 0.001.

## Results

### Changes in the Chl accumulation in petals resulted in the degreening of *P. suffruticosa* ‘LMYY’ flowers during blooming

**Fig. 1:**
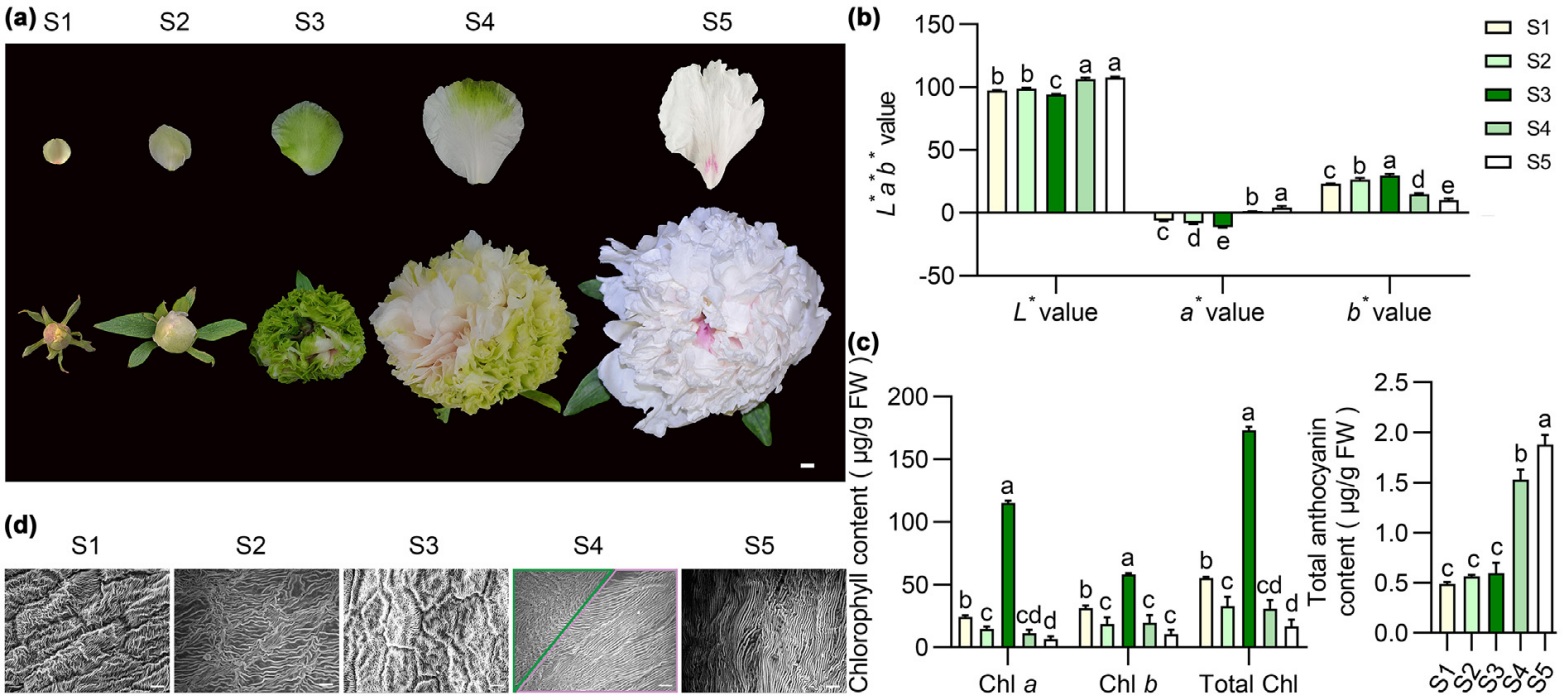
**Flower color parameters, Chl and anthocyanin accumulation patterns in *P. suffruticosa* ‘LMYY’ petals at five developmental stages. a)** The petal phenotypes at five developmental stages, scale bar = 1 cm. **b – c)** CIEL*a*b* color parameter, and the content of Chl and anthocyanin over five stages. Values are presented as the means ± SD from three biological replicates. Different letters above each bar denote significant differences according to one-way ANOVA with Tukey’s multiple comparisons test (*P* < 0.05). **d)** The adaxial epidermal cells in the middle of the petal over five stages, yellow lines represent the cell boundary, scale bars = 10 μm.

*P. suffruticosa* ‘LMYY’ is a popular cultivar with green flowers. Its flower developmental process was divided into five stages: S1, petals with yellow-green color at tight flower bud stage; S2, petals were light green at swollen flower bud stage; S3, petals were deep green and began to degreen from the petal base at initiating blooming stage; S4, petals degreened to pale pink at the cup-blooming stage; S5, petals were pale pink at blooming stage (Fig. 1a). The colors were evaluated, and the L* value decreased from 97.54 (S1) to 94.21 (S3) and then increased to 106.58 (S4) and 107.61 (S5). The a* value was negative and decreased from −6.01 (S1) to −11.12 (S3), and increased from 1.25 (S4) to 4.31 (S5), while the b* value increased from 23.30 (S1) to 29.86 (S3) then decreased to 14.69 (S4), and then reached the minimum value 10.43 (S5). The changes in the above parameters were consistent with the degreening of petals during blooming (Fig. 1b).

Chl is the primary pigment determining the greenness of petals. To further analyze the degreening pattern of ‘LMYY’ petals during blooming, the Chl levels were measured. The contents of Chl *a*, Chl *b*, and total Chl followed similar patterns, which first decreasing from S1 to S2, then increasing rapidly to the maximum values at S3, which were 115.20 (Chl *a*), 57.96 (Chl *b*), and 173.16 (total Chl) μg/g FW, respectively. After S3, the levels decreased, reaching minimum values at S5, which were 6.37 (Chl *a*), 10.43 (Chl *b*), and 16.80 (total Chl) μg/g FW, respectively (Fig. 1c). We found the total anthocyanin levels were very low but gradually increased during flower development and blooming (S1: 0.49, S2: 0.56, S3: 0.60 and S4: 1.53 μg/g FW), reaching a maximum value of 1.89 μg/g FW at S5 (Fig. 1c), consistent with the petal color.

### Epidermal cell differences were observed in degreening petals during blooming

Color perception by the human eye depends on the light reflection of diverse-shaped petal epidermal cells. In this study, epidermal cell shapes across the five different developmental stages were compared during flower development and blooming. Most significant differences were identified in adaxial epidermal cells (Fig. 1d). The variance of striation packing on epidermal cells was associated with Chl content. At S3, when the petals had the highest Chl content, the striations of epidermal cells were most densely packed, which is the opposite at S5 with the lowest Chl content. Notably, two types of epidermal cells with different striation packing patterns were observed in petals with green and pale pink colors at S4. The difference in epidermal cell shape was beneficial to understanding petal color changes.

### Petal transcriptome analysis and unigenes identified associated with Chl degradation

Combined with the change in Chl content and epidermal cell shape of petals, transcriptome analysis was conducted on petals at three critical developmental stages (S1, S3, and S5) to characterize candidate genes related to Chl degradation. A total of 32.02 Gb of clean reads were obtained with 94.48 to 94.78% Q30 value (average 94.60%) and GC contents of 45.01 to 46.87% (average 45.73%) (Table S2). Moreover, 51,535 unigenes were assembled with an N50 of 1,443 bp.

Analysis of DEGs was performed between every two developmental stages (S1 *vs* S3, S3 *vs* S5, and S1 *vs* S5). A total of 4,643 up- and 4,595 down-regulated genes were detected among the three comparison groups, including 3,037 (1,419 up and 1,618 down), 2,996 (1,652 up and 1,344 down), and 3,205 (1,572 up and 1,633 down) DEGs in S1 *vs* S3, S3 *vs* S5, and S1 *vs* S5, respectively (Fig. S1). A total of 3,754 up- and 4,412 down-regulated DEGs were enriched via GO functional classification. The most abundant DEGs were annotated in metabolic process (biological process), cell part (cellular component), and binding (molecular function), respectively (Fig. S2). DEGs were enriched in photosynthesis-antenna proteins and photosynthesis in both S1 *vs* S3 and S3 *vs* S5 (Fig. S3), indicating some differences in photosynthesis during flower development and blooming. Furthermore, both DEG sets were rich in anthocyanin and flavonoid biosynthesis in S1 *vs* S5 and S3 *vs* S5 (Fig. S3), which indicated that anthocyanin and flavonoid biosynthesis occurred in flower blooming.

**Fig. 2:**
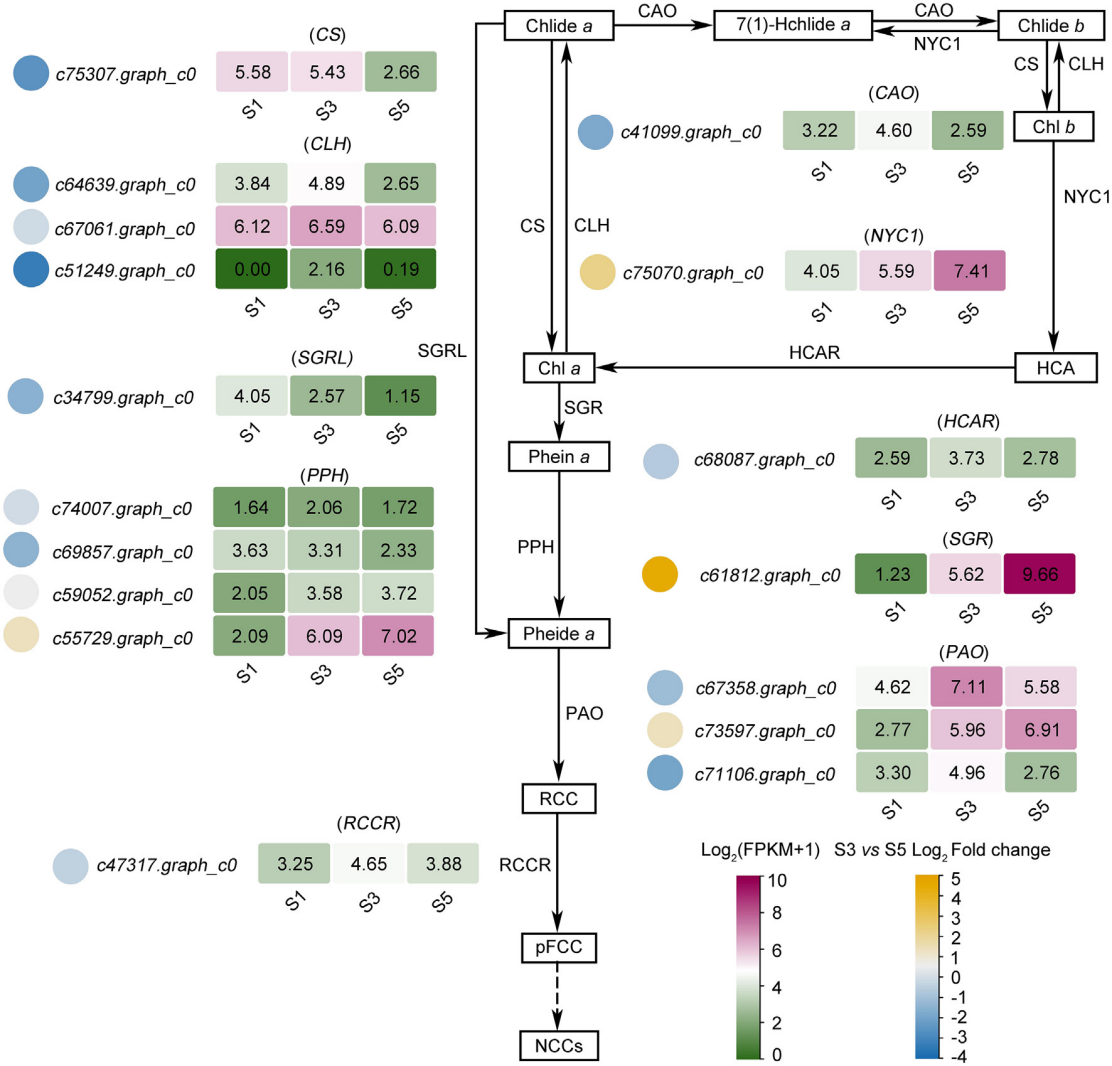
Transcriptome profiles of genes putatively involved in the Chl degradation pathway at three developmental stages of *P. suffruticosa* ‘LMYY’ petals. The heatmap shows the gene expression patterns. The circles on the left of the genes represent the log_2_fold change value from S3 to S5, and the round rectangles on the right of the genes represent the log_2_(FPKM+1) value. Chl, chlorophyll. Phein, pheophytin. Pheide, pheophorbide. Chlide, chlorophyllide. SGR, Mg-dechelatase STAY-GREEN. PPH, pheophytinase. PAO, pheophorbide *a* oxygenase. CLH, chlorophyllase. CAO, chlorophyllide an oxygenase. NYC1, non-yellow coloration 1. CS, chlorophyll synthase. HCAR, 7-hydroxymethyl chlorophyll *a* reductase. RCCR, red chlorophyll catabolic reductase.

The Chl degradation is crucial for petal degreening. To further explore the mechanism of Chl degradation during blooming from S3 to S5, the expression patterns of core genes, including *Chlorophyllide an oxygenase* (*CAO*), *Non-yellow coloration 1* (*NYC1*), *Chlorophyll synthase* (*CS*), *CLH*, *7-hydroxymethyl chlorophyll a reductase* (*HCAR*), *SGR*, *SGRL*, *PPH*, *PAO*, and *Red chlorophyll catabolic reductase* (*RCCR*) were analyzed (Fig. 2 and Table S3). *CAO* (*c41099.graph_c0*), *CS* (*c75307.graph_c0*), *CLH* (*c64639.graph_c0*, *c67061.graph_c0*, *c51249.graph_c0*), *HCAR* (*c68087.graph_c0*), *SGRL* (*c34799.graph_c0*), and *RCCR* (*c47317.graph_c0*) were down-regulated, while *NYC1* (*c75070.graph_c0*) and *SGR* (*c61812.graph_c0*) were up-regulated. Two *PPHs* (*c74007.graph_c0* and *c69857.graph_c0*) were down-regulated, and the others (*c59052.graph_c0* and *c55729.graph_c0*) exhibited the opposite trend during petal degreening. Additionally, two *PAOs* (*c67358.graph_c0* and *c71106.graph_c0*) were down-regulated, and only the *c73597.graph_c0* expression level increased. CLH is the initial and rate-limiting enzyme of Chl degradation [18]. Among three *CLH*s, *c51249.graph_c0* had the most significant change from S3 to S5, suggesting it might play an important role in Chl degradation in petals. We evaluated the expression pattern of *PsCLH1* by qRT-PCR, which was similar to the RNA-seq result (Fig. S4a). Therefore, we focused on *c51249.graph_c0* (*PsCLH1*) in further experimentation.

**Fig. 3:**
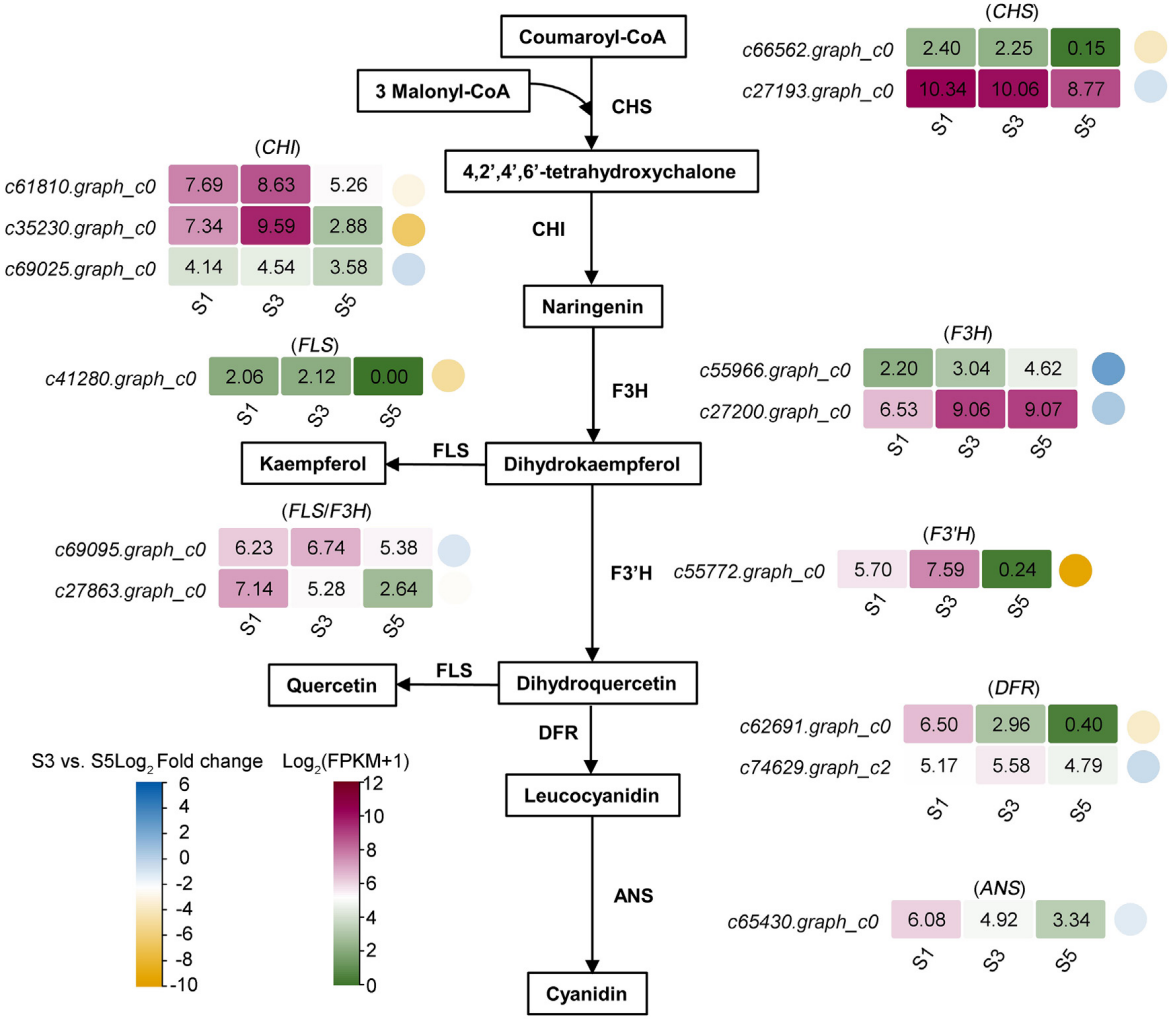
Transcriptome profiles of genes putatively involved in the anthocyanin biosynthetic pathway at three developmental stages of *P. suffruticosa* ‘LMYY’ petals. The heatmap shows the gene expression patterns. The circles on the left of the genes represent the log_2_fold change value from S3 to S5, and the round rectangles on the right of the genes represent the log_2_(FPKM+1) value. CHS, chalcone synthase. CHI, chalcone isomerase. F3H, flavanone 3-hydroxylase. F3′H, flavonoid 3′-hydroxylase. DFR, dihydroflavonol 4-reductase. ANS, anthocyanin synthase. FLS, flavonol synthase.

The total anthocyanin content was increased in ‘LMYY’ petals during blooming (Fig. 1c). In the anthocyanin biosynthetic pathway, malonyl-CoA and 4-coumarpyl CoA are condensed by chalcone synthase (CHS) to produce chalcones, which are converted to cyanidin sequentially by chalcone isomerase (CHI), flavanone 3-hydroxylase (F3H), flavonoid 3′-hydroxylase (F3′H), dihydroflavonol 4-reductase (DFR), and anthocyanin synthase (ANS) [37]. To further investigate the anthocyanin metabolism mechanism during blooming, the expression patterns of *CHS*, *CHI*, *F3H*, *F3′H*, *Flavonol synthase* (*FLS*), *DFR*, and *ANS* were analyzed (Fig. 3. and Table S4). The expression level of *F3H* (*c27200.graph_c0*) increased from S1 to S5, consistent with anthocyanin accumulation. However, most genes, including *CHI* (*c61810.graph_c0*, *c35230.graph_c0*, and *c69025.graph_c0*), *FLS*/*F3H* (*c69095.graph_c0*), *F3′H* (*c55772.graph_c0*), and *DFR* (*c74629.graph_c2*) were up-regulated from S1 to S3, peaking at S3, and down-regulated during blooming (S3 to S5), suggesting that S3 was the key point when petal color changed from green to pale pink.

### *PsCLH1* regulated the Chl accumulation and promoted the degreening of ‘LMYY’ petals

**Fig. 4:**
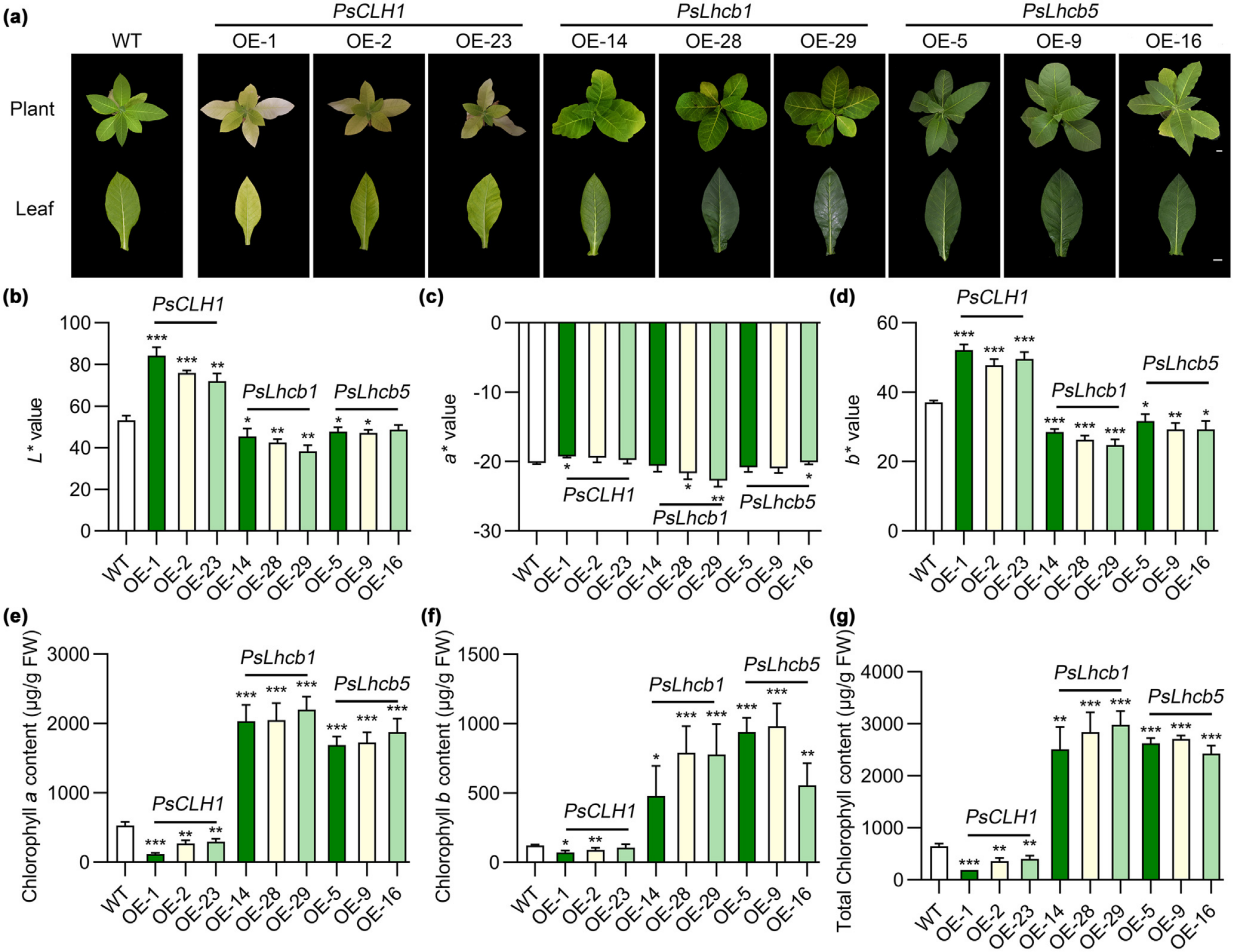
**Overexpression of *PsCLH1*, *PsLhcb1*, and *PsLhcb5* affected Chl accumulation in tobacco leaves.a)** Phenotype of WT plants and OE lines, scale bars = 1 cm. **b – d)** CIEL*a*b* color parameter of WT and OE leaves. **e – g)** The Chl levels of WT and OE leaves. Values are presented as means ± SD from three biological replicates. Asterisks indicate significantly different values compared to the WT control (*, *P <* 0.05, **, *P <* 0.01, and ***, *P <* 0.001, two-tailed student’s *t*-test).

To further determine the function of *PsCLH1 in vivo*, we ectopically overexpressed *PsCLH1* in tobacco (*N. tabacum*), and three transgenic lines (OE-1, OE-2, and OE-23) with high expression levels of *PsCLH1* were selected for further experiments. The leaves of wild-type (WT) plants were greener than those of *PsCLH1*-OE plants (Fig. 4a-d), consistent with the Chl content of leaves in WT and *PsCLH1*-OE lines (Fig. 4e-g). There were significant differences between *PsCLH1*-OE lines and WT plants in growth status. The plant height, the number of leaves, crown width, the number of fruits per plant, and single fruit weight all decreased in *PsCLH1*-OE lines compared to WT plants (Fig. S5), suggesting that WT plants growth vigor is stronger than *PsCLH1*-OE lines.

**Fig. 5:**
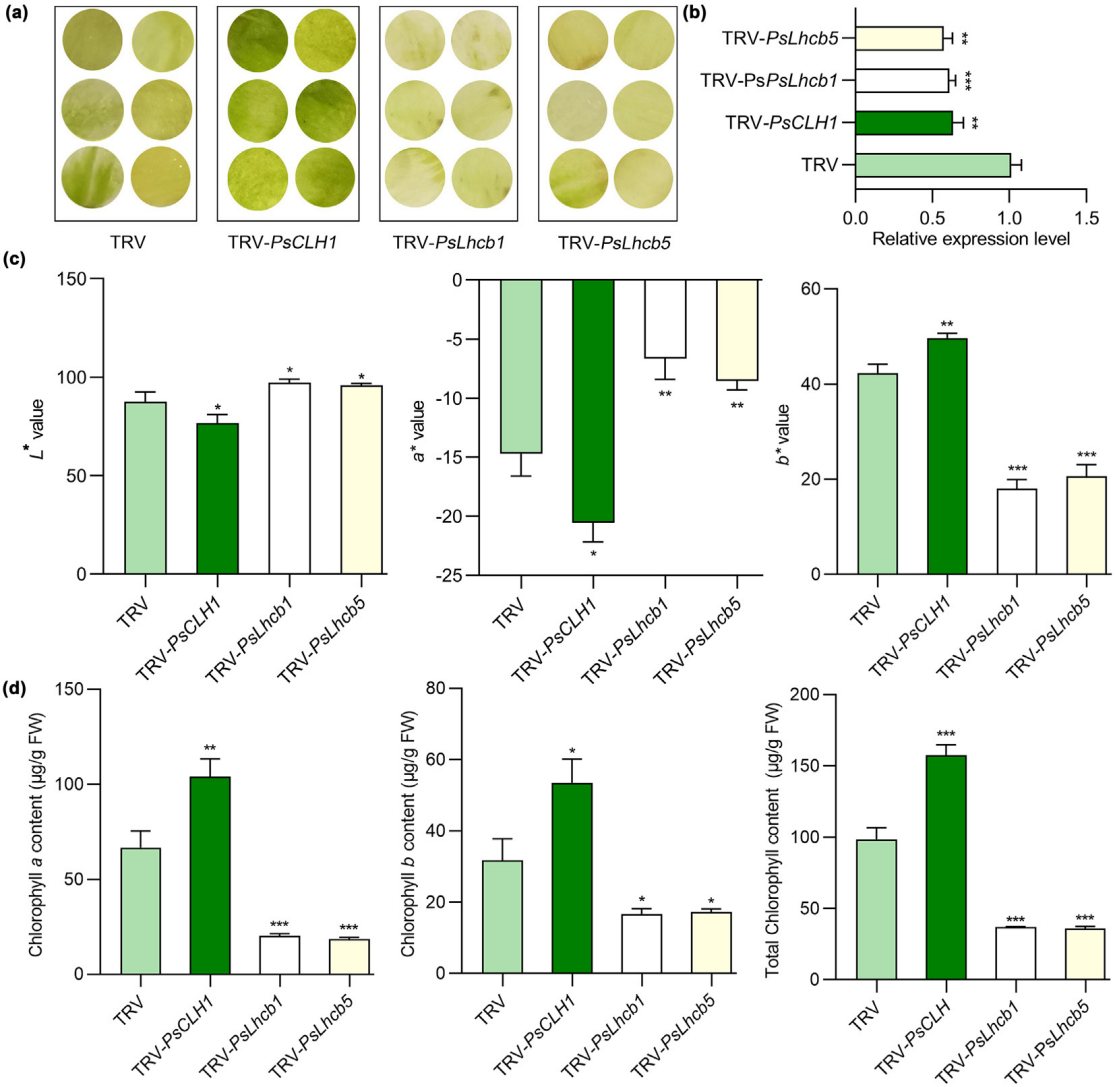
**Silencing of *PsCLH1*, *PsLhcb1*, and *PsLhcb5* affected Chl accumulation in *P. suffruticosa* ‘LMYY’ petals. a)** Silencing of *PsCLH1*, *PsLhcb1*, and *PsLhcb5* changed the color of flower petals. Representative images of TRV control and silenced petals were taken ten days after infiltration. **B)** qRT-PCR analysis of *PsCLH1*, *PsLhcb1*, and *PsLhcb5* in TRV control and silenced flower petals. **c – d)** CIEL*a*b* color parameter (c) and Chl content (d) in TRV control and silenced flower petals. Values are presented as the means ± SD from three biological replicates. Asterisks indicate significantly different values, compared to the TRV control (*, *P <* 0.05, **, *P <* 0.01, and ***, *P <* 0.001, two-tailed student’s *t*-test).

To verify its role in regulating Chl content, we silenced *PsCLH1* using VIGS technology in ‘LMYY’ petals. The *PsCLH1*-silenced petals were greener than that of the TRV control (Fig. 5a), and the expression level of *PsCLH1* in the silenced line was significantly reduced by 37.62 % compared to the TRV control (Fig. 5b). The L* and a* values of the *PsCLH1*-silenced line (L*76.74, a*-20.56) were lower than the TRV control (L*87.60, a*-14.71), while the b* value demonstrated an opposite trend to the L* and a* values (Fig. 5c), consistent with the phenotypic results (Fig. 5a). The total Chl content of *PsCLH1*-silenced petals (157.60 µg/g FW) was significantly increased compared to the TRV control (98.50 µg/g FW) (Fig. 5d). These findings indicated that *PsCLH1* decreased the Chl content and promoted the degreening of ‘LMYY’ petals.

### Expression patterns and sequence analysis of *PsLhcs* in ‘LMYY’

As a coordinator of antenna pigments in the light-harvesting complex across both PS I and II, Lhcs play important roles in regulating Chl levels [21], [38]. We also placed emphasis on the functional characterization of Lhcs in ‘LMYY’ petal degreening. Seven *PsLhcs* were identified in the ‘LMYY’ petals transcriptome, and all were down-regulated significantly from S3 to S5 (Table S5), suggesting that *PsLhcs* played an important role in ‘LMYY’ petal degreening. We focused on *c72784.graph_c0* and *c27170.graph_c0*, with the most significant expression level change in degreening petals (S3 *vs* S5). Moreover, we explored the expression patterns of *c72784.graph_c0* and *c27170.graph_c0* by qRT-PCR, which were similar to the RNA-seq results (Fig. S4b and c).

**Fig. 6:**
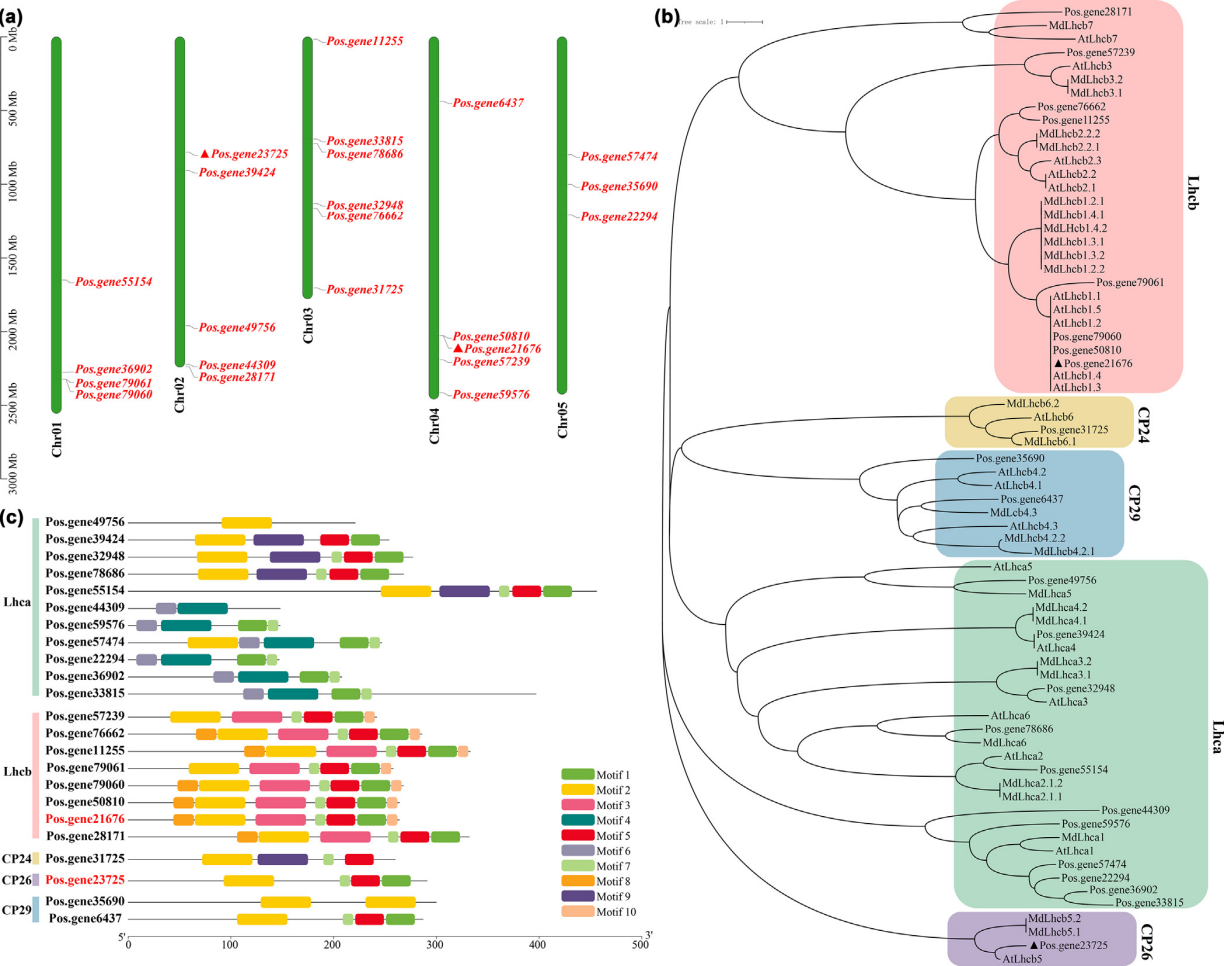
**The characterization of *Lhcs*. a)** Chromosomal location of *Lhcs* on *P. ostii* chromosomes. **b)** The phylogenetic tree of Lhcs from *A. thaliana*, *M. domestica*, and *P. ostii*, constructed using the Neighbor-Joining method based on the *p*-distance model, involving 71 amino acid sequences and pairwise deletion. **c)** The distribution of conserved motifs in Lhcs. Putative motifs are shown as different colored boxes; refer to Supplemental Table S6 for details.

We further identified all the members of the *PsLhc* family genes through BLAST alignment in the *P. ostii* genome [39], and 23 *PoLhcs* were obtained. There were 4, 5, 6, 5, and 3 *PoLhcs* located from chromosomes 1 through 5, respectively, among which, *Pos.gene23725* (*c27170.graph_c0*) and *Pos.gene21676* (*c72784.graph_c0*) on chromosomes 2 and 4, respectively (Fig. 6a). To understand the evolutionary relationship and grouping characteristics, a phylogenetic tree was produced from 23 PoLhcs together with 21 and 27 Lhc proteins from *A. thaliana* and *M. domestica*, respectively (Fig. 6b). The 23 PoLhcs were divided into five subfamilies, including Lhca, Lhcb, CP24, CP26, and CP29. Within each subfamily, Lhca had the highest number of members (11), followed by Lhcb (8), CP29 (2), CP26 (1), and CP24 (1). Among them, *Pos.gene23725* and *Pos.gene21676* belonged to CP26 and Lhcb subfamilies, respectively, and the nearest proteins were AtLhcb5 and AtLhcb1, respectively. A total of ten motifs were identified, and these conserved motif lengths varied from 11 to 50 amino acids (Fig. 6c, Table S6). According to these results, *Pos.gene23725* and *Pos.gene21676* were named *PsLhcb5* and *PsLhcb1*, respectively.

### Both *PsLhcb1* and *PsLhcb5* regulated Chl accumulation and repressed the degreening of ‘LMYY’ petals

To identify the function of *PsLhcb1* and *PsLhcb5 in vivo*, we ectopically overexpressed these two genes in tobacco. In the subsequent experiments, three transgenic lines with high expression levels of *PsLhcb1* (OE-14, OE-28, and OE-29) and *PsLhcb5* (OE-5, OE-9, and OE-16) were selected. The leaves of both *PsLhcb1-* and *PsLhcb5*-OE lines were greener than WT plants (Fig. 4a − d), and the Chl levels of leaves in *PsLhcb1* and *PsLhcb5*-OE lines were significantly higher than that of WT plants (Fig. 4e − g), consistent with the leaf color. Moreover, the plant height, the number of leaves, crown width, the number of fruits per plant, and single fruit weight all increased sharply in *PsLhcb1* and *PsLhcb5*-OE lines compared to WT plants (Fig. S5), suggesting the *PsLhcb1* and *PsLhcb5*-OE growth vigor was higher than WT plants. *PsLhcb1* and *PsLhcb5* also increased the yield of tobacco.

To verify their roles in regulating Chl content, we silenced *PsLhcb1* and *PsLhcb5* using VIGS technology in ‘LMYY’ petals. The TRV control petals were greener than *PsLhcb1* and *PsLhcb5*-silenced lines (Fig. 5a), and the expression levels of *PsLhcb1* and *PsLhcb5* in silenced lines were significantly reduced by 39.60% and 43.56% relative to TRV control, respectively (Fig. 5b). The L* and a*values of *PsLhcb1* (L*97.37, a*-6.65) and *PsLhcb5*-silenced lines (L*95.98, a*-8.54) were higher than the TRV control (L*87.60, a*-14.71), and the b* value demonstrated an opposite trend to the L* and a* values (Fig. 5c), consistent with the phenotypic results. The total Chl content of *PsLhcb1* (36.94 µg/g FW) and *PsLhcb5*-silenced petals (35.86 µg/g FW) was sharply reduced compared to the TRV control (98.50 µg/g FW) (Fig. 5d). These findings suggested that *PsLhcb1* and *PsLhcb5* increased the Chl content and repressed the degreening of ‘LMYY’ petals.

### The co-expression network and promoter analysis of *PsCLH1, PsLhcb1*, and *PsLhcb5*

**Fig. 7:**
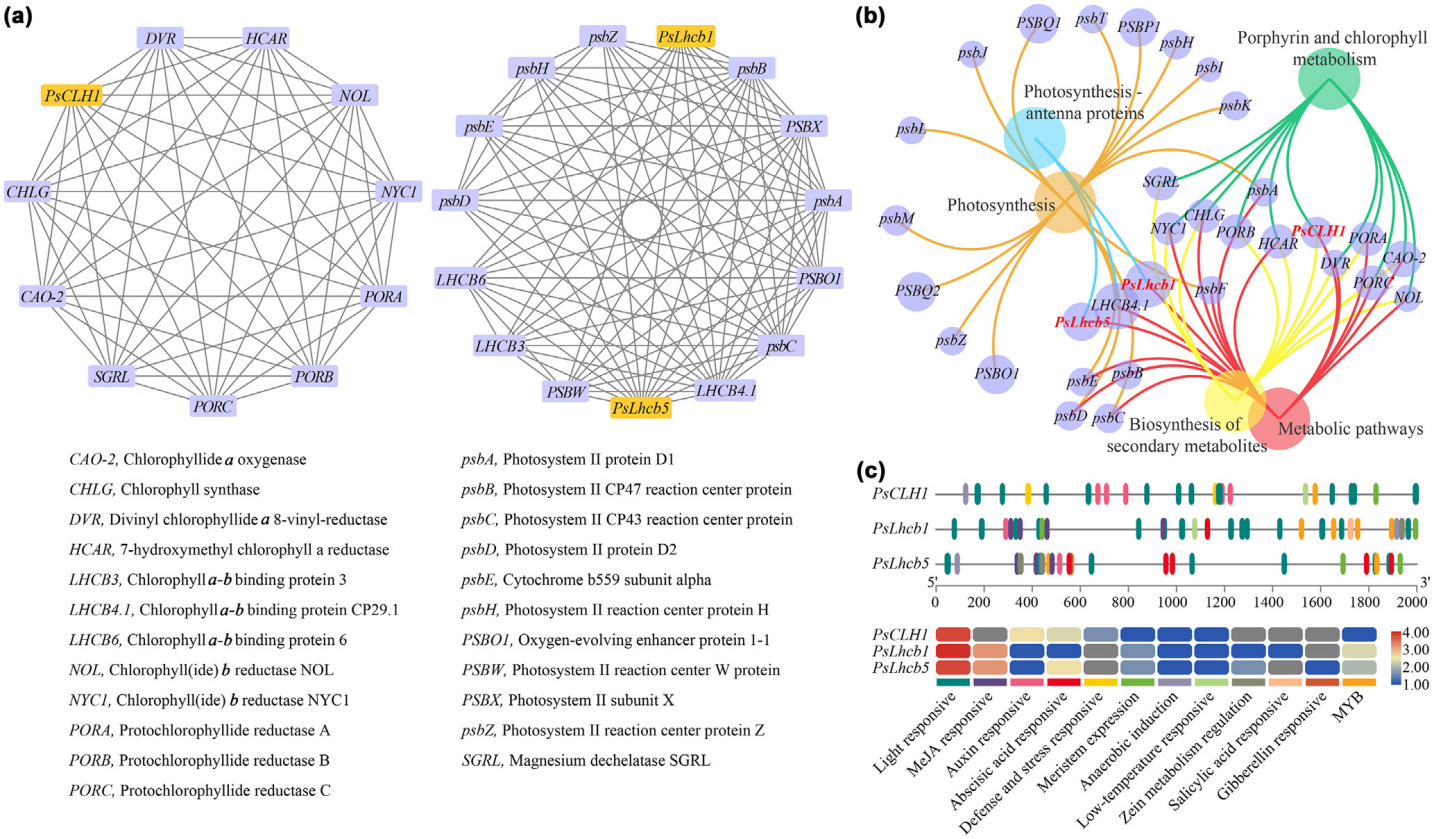
**The co-expression network of *PsCLH1*, *PsLhcb1*, *PsLhcb5*, and *cis*-elements of promoters. a)**. The co-expressed genes with *PsCLH1*, *PsLhcb1*, and *PsLhcb5.***b)** The co-expressed genes enriched in KEGG pathways with *PsCLH1*, *PsLhcb1*, and *PsLhcb5.***c)** The distribution and enrichment of *cis*-elements of *PsCLH1*, *PsLhcb1*, and *PsLhcb5* promoters.

Given the functional relationship between *PsCLH1*, *PsLhcb1*, and *PsLhcb5*, a co-expression network of *PsCLH1*, *PsLhcb1*, and *PsLhcb5* was conducted. The 23 co-expressed genes were enriched in photosynthesis, porphyrin and Chl metabolism, photosynthesis-antenna proteins, biosynthesis of secondary metabolites, and metabolic pathways, suggesting their coordinated function in petal greenness maintenance. Genes co-expressed with *PsCLH1* included those encoding enzymes associated with the Chl metabolism pathway like Chl synthase (CHLG), Protochlorophyllide reductase A (PORA) and PORB, NYC1, Chlorophyll(ide) *b* reductase (NOL), HCAR, and SGRL. The co-expressed genes with *PsLhcb1* and *PsLhcb5* are involved in photosystem responses, including genes encoding photosystem proteins, namely Photosystem II protein D1 (psbA) and psbB, and other Chl-binding proteins (eg. LHCB3 and LHCB6) (Fig. 7a and b). Numerous light-responsive *cis*-elements have been identified in the promoter sequences of *PsCLH1*, *PsLhcb1*, and *PsLhcb5*, demonstrating their critical role in the photosynthetic pathway (Fig. 7c). Additionally, there were methylated jasmonic acid (MeJA), auxin-responsive, and MYB binding elements on their promoters, suggesting the potential regulation by phytohormones and MYB transcription factors.

**Fig. 8:**
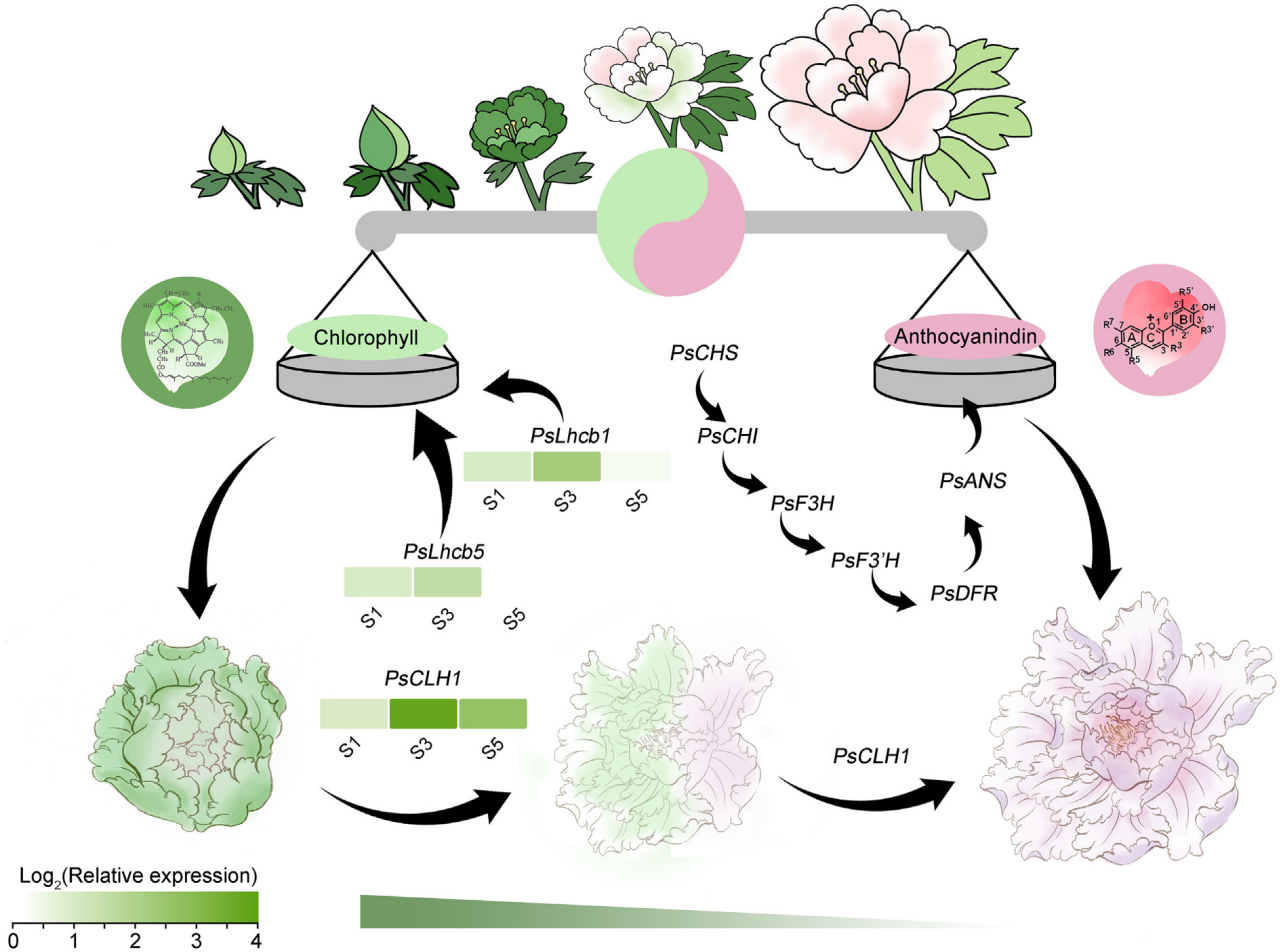
A putative model for petal degreening regulated by the balance of Chl degradation and anthocyanin biosynthesis. The relationship between the two processes is like a scale, where the tilted side of the balance presents a color in that direction. *PsCLH1* promoted degreening, while *PsLhcb1* and *PsLhcb5* repressed the degreening of petals. S3 is the critical juncture when anthocyanin biosynthesis pathway genes (*CHI*, *F3′H*, *DFR*, etc.) demonstrate high expression levels at the onset of Chl degradation.

Collectively, our findings suggest a model for regulating the balance of Chl and anthocyanin metabolism in ‘LMYY’ green petals during blooming (Fig. 8). The relationship between the two processes is like a scale, where the tilted side of the balance presents a color. The expression of *PsLhcb1* and *PsLhcb5* regulated the accumulation of the Chl and promoted petal greening. The expression of *PsCLH1* accelerated the degradation of the Chl and promoted petal degreening. S3 acts as the critical point, when anthocyanin biosynthesis pathway genes (*CHI*, *F3′H*, *DFR*, etc.) demonstrate high expression levels, while Chl degradation genes *PsCLH1* and *PsLhcs* cooperate to regulate Chl accumulation and the degreening of *P. suffruticosa* ‘LMYY’ petals during blooming.

## Discussion

Flower color is a critical trait in flowering plants, and green flowers are very rare in nature. Due to the lack of a proper model, associated research remains limited. We utilized the *P. suffruticosa* ‘LMYY’ as a research model to uncover the molecular mechanism of petal degreening. During the flower development and blooming, the color of the petals changed from yellow-green (S1) to deep green (S3), then the green faded to pale green (S4), and finally pale pink (S5), consistent with the levels of Chl and anthocyanin. Three key DEGs were functionally characterized through overexpression in tobacco plants and silencing in ‘LMYY’ petals. A model was proposed to regulate the balance of Chl and anthocyanin metabolism in ‘LMYY’ green petals throughout blooming. This study will illuminate our understanding of flower color evolution and offer a strategy for cultivar breeding with innovative flower colors.

The morphology and structure of petal epidermal cells significantly influence the absorption and reflection of light, which affects flower color. Pigmentation can be affected by the shape of epidermal cells. During flowering, the conical shape of the cells near the epidermis results in increased light absorption by anthocyanins, enhancing petal color [40], [41]. The morphology alterations of epidermal cells across the five distinct green flowering periods were observed, suggesting that cells with darker pigmentation exhibit a greater number of folds and are more densely packed than their lighter counterparts. At the S4 stage, in which the two colors coexist, the morphological differences in the epidermal cells are particularly evident. The causality for green petals in tree peonies requires further characterization, as limited studies have been reported in tree peonies to date. In other plants, similar findings were observed, the epidermal cells of *Zantedeschia hybrida* petals with flatter epidermal cells caused more light to reflect and made the petal color lighter [42]. Epidermal cell shapes in *Dendrobium* flowers may contribute to petal color differences, and the conical degree of the epidermal cells on the darker lip is higher than that in lighter petals [43], [44]. The epidermal cells of the *Antirrhinum majus* petals changed from conical to flat, and the color of the petals also changes from dark to light [45]. Structural color generated by epidermal structure may affect signaling to pollinating insects via colorful petals and patterning [41], however, the role of the petal cell structure of green petals in functioning requires further research. The above results quantify the color phenotype of tree peony green flowers and establish a foundation for further study of underlying color-presenting mechanisms and breeding of innovative varieties. In the future, genes associated with epidermal cell shape should be screened and identified to understand their function in influencing *P. suffruticosa* flower colors.

CLH is the initial and rate-limited enzyme in Chl degradation, which hydrolyzes Chl to produce Chlide and phytol [18], [46]. In many plant’ species, such as *Piper betle* [47] and *Citrus limon* [48], the activity of CLH is negatively correlated with Chl content and directly governs the degradation of the Chl. However, the conducted studies have stated conflicting results in this regard. In *Arabidopsis*, Chl degradation is not suppressed in *clh1* and *clh2* single and double knockout lines, which indicates that CLH is not necessary for Chl degradation [49], [50]. Functional characterization indicated that the up-regulated expression of *PsCLH1* accelerated Chl degradation, suggesting it was essential for this process in ‘LMYY’ green petals. However, the expression level of *PsCLH* was positively correlated with the Chl content, reaching their highest levels at S3. Based on several researches, CLH performed a function as a rate-limiting enzyme in Chl degradation, controlled by posttranslational regulation, resulting in its latent function [18]. Whether and how posttranslational regulation of PsCLH occurs in ‘LMYY’ green petals requires further study.

*Lhcs* are widely involved in widespread biological processes in higher plants, such as leaf, flower, and fruit growth and development. Members of the *Lhcs* family have been identified in various plants, such as *Jatropha curcas* (15) [36], *Vitis vinifera* (20) [23], *M. domestica* (27) [36], and *Gossypium hirsutum* (55) [51], exhibiting significant differences in the number of *Lhcs* family members. However, no relevant studies on tree peonies have been previously reported. Genome-wide identification of *Lhcs* family members in tree peonies was conducted, and 23 were obtained and located across five chromosomes, among which *PsLhcb1* and *PsLhcb5* were functionally characterized. Transgenesis in tobacco and silencing in ‘LMYY’ petals indicated these two genes increased the Chl content and affected degreening. Recently, *Lhcs* members were found to promote Chl accumulation and control leaf greenness in *Camellia sinensis* (*LHCII*, *CSA035910*) [52], *Lagerstroemia indica* (chlorophyll *a*/*b* binding protein gene, *CAB1*) [53], *G. hirsutum* (*Lhcb2.3*) [51], and *Actinidia* (*Lhcb3.1*/*3.2*) [38], but for the first time, *Lhcs* has been functionally characterized in green petals. After enhancing the expression of *PsLhcb1* and *PsLhcb5* in tobacco, growth vigor was more robust than that in WT plants, and the fruit yield was increased, consistent with the results in *Oryza sativa* [54], [55]. To enhance light energy capture ability and increase yield per unit of land area, *Lhcs* can be employed for germplasm innovation against yield limitation in the future.

We found that *PsCLH1* co-expressed with *NYC1*, *NOL*, *HCAR*, and *SGRL*, indicating these genes may also play an important role in ‘LMYY’ petal degreening. Mutations in *NYC1*, *NOL*, *HCAR*, and *PAO* could cause the stay-green phenotype [56], [57], [58], [59], demonstrating that the SGRL protein plays a vital role in the early phase of Chl degradation [60]. Additionally, Chl is associated with Chl-binding proteins in the PSI and PSII complexes [61] and accumulates in tissues where PSI and PSII are produced. We found *Lhcs* co-expressed with *Psb* genes for diverse subunits of PSII. However, the function of the genes mentioned above in *P. suffruticosa* petals remains unknown and must be further studied. Enzyme genes and transcriptional factors associated with petal greening suggest that increasing *CONSTANS-like 16* (*COL16*) or *SUPPRESSOR OF OVEREXPRESSION OF CO 1* (*SOC1*) resulted in green petals in *Arabidopsis*, *Petunia*, and tobacco [62], [63]. TEOSINTE BRANCHED1/CYCLOIDEA/PCF4 (TCP4) controlled petal greenness by directly binding to the promoters of *PORB*, *DIVINYL REDUCTASE* (*DVR*), and *SOC1* [15]. In our study, many *cis*-elements were identified in the promoter sequences of *PsCLH*, *PsLhcb1*, and *PsLhcb5*. Therefore, research on transcriptional control would be an interesting topic in the future. Most studies have focused on the transcriptional control of anthocyanin metabolism, and future studies on the spatial and temporal expression regulation of genes associated with Chl and anthocyanin metabolism pathways would be meaningful to comprehend the genetic basis for balanced organ-specific Chl and anthocyanin accumulation. The study provides a theoretical basis for the regulation and molecular breeding of tree peony flower color, offering insights into the cultivation of new varieties of green flowers in other ornamental plants. It is expected to facilitate the creation of greater economic value within the flower industry. Further research will be conducted into the chemical and molecular biological mechanisms underlying green flower color, with the aim of generating additional industrial and scientific value.

## Conclusions

In this study, we identified petal color changes from green to pale pink that were consistent with the levels of Chl and anthocyanin. Three key genes were identified in Chl accumulation, among which *PsCLH1* promoted degreening, while *PsLhcb1* and *PsLhcb5* repressed degreening of *P. suffruticosa* ‘LMYY’ petals. Our findings elucidate the molecular mechanisms of petal green maintaining, which provide insights into underlying flower color evolution and breeding cultivars with innovative flower colors.

## Declaration of competing interest

The authors declare that they have no known competing financial interests or personal relationships that could have appeared to influence the work reported in this paper.
