# Supplementary material for: Chlorophyllase (PsCLH1) and light-harvesting chlorophyll a/b binding protein 1 (PsLhcb1) and PsLhcb5 maintain petal greenness in Paeonia suffruticosa ‘Lv Mu Yin Yu’
Source: J Adv Res. 2024 Sep 3;73:173–85. doi: 10.1016/j.jare.2024.09.003 (PMC12225939; doi:10.1016/j.jare.2024.09.003)
Supplement: Supplementary Data 1 [file mmc1.pdf]

Supplementary figures

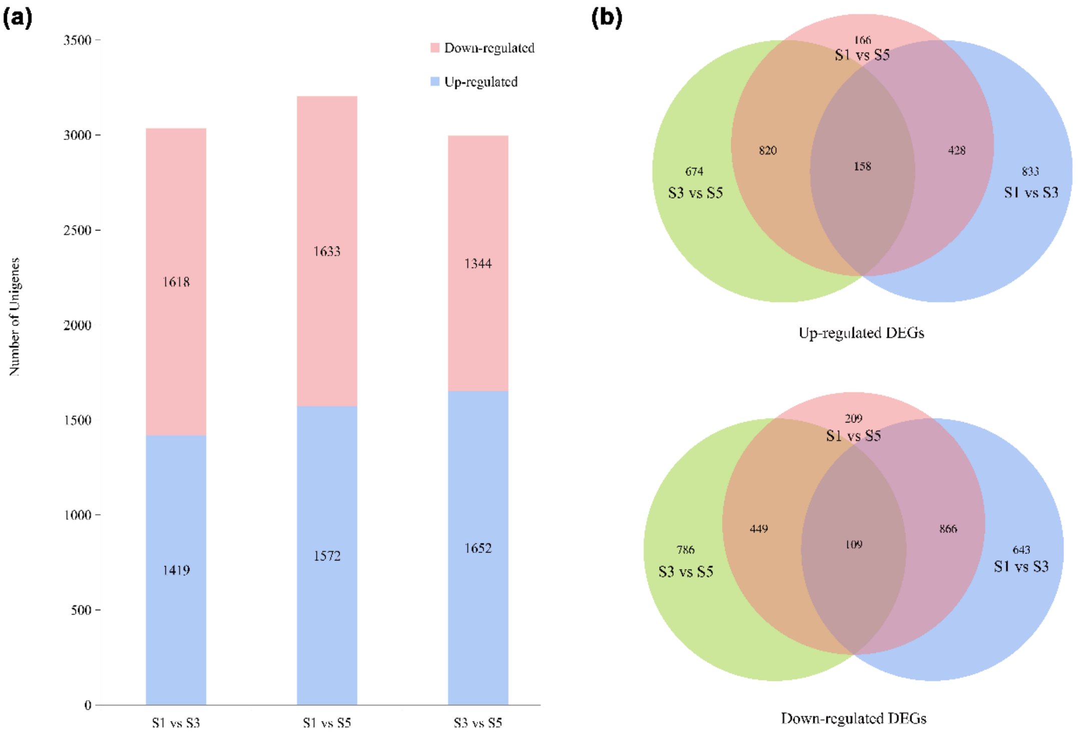

Fig. S1 Statistics of DEGs. a) The number of up-and down-regulated DEGs in the pairwise group. **b)** Venn diagram of up- and down-regulated DEGs. The $|\log_2(\text{fold change})| \geq 1$ and FDR ≤ 0.001 were the threshold for determining significant DEGs.

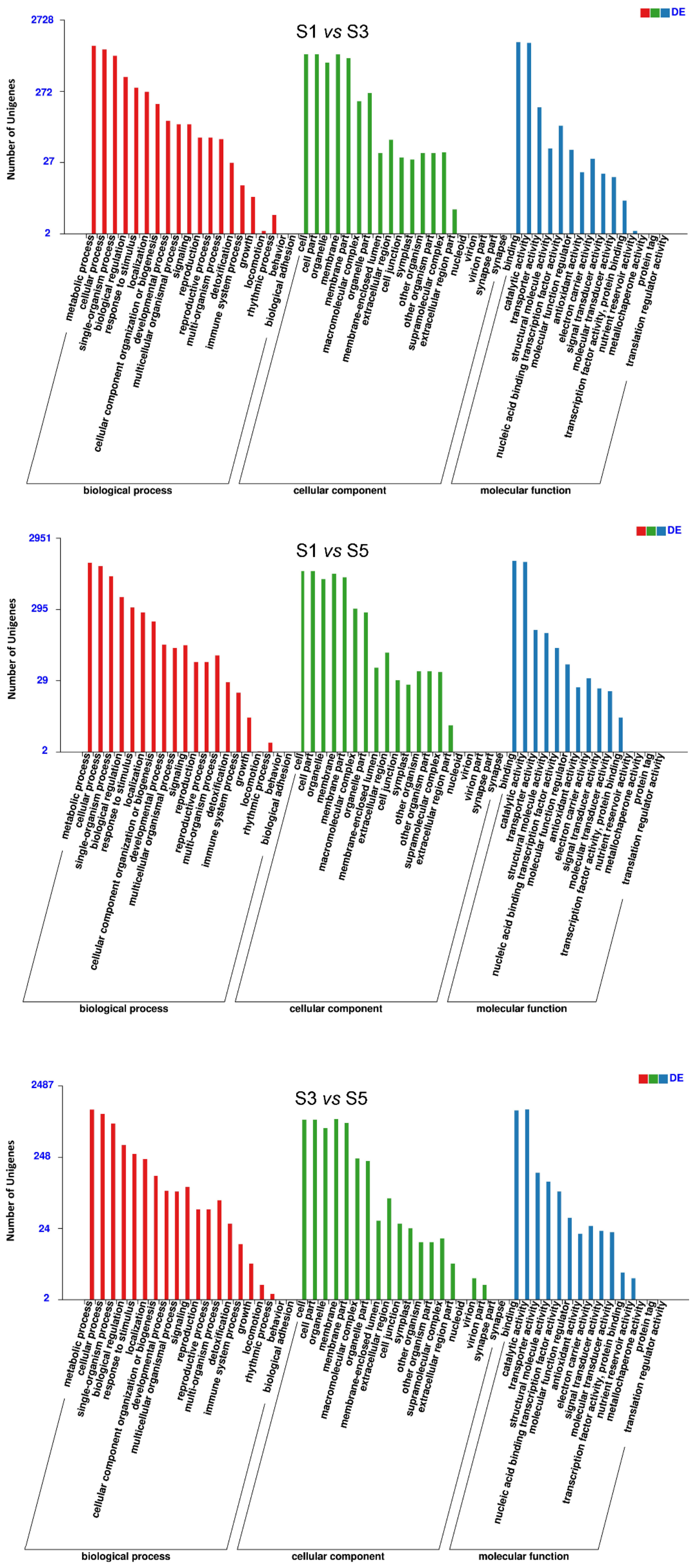

Fig. S2 GO classification of the DEGs between S1 *vs* S3, S1 *vs* S5, and S3 *vs* S5, respectively.

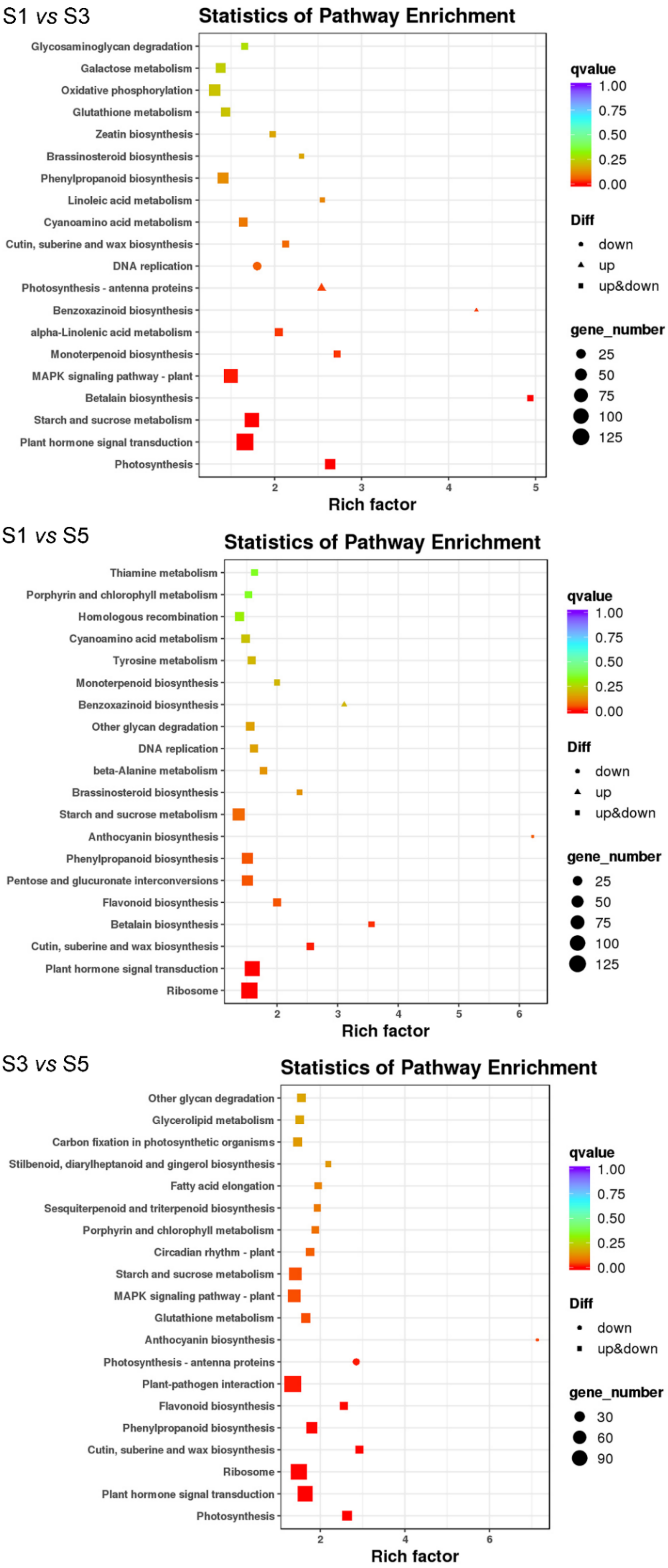

Fig. S3 KEGG enrichment analysis of DEGs between S1 *vs* S3, S1 *vs* S5, and S3 *vs* S5, respectively.

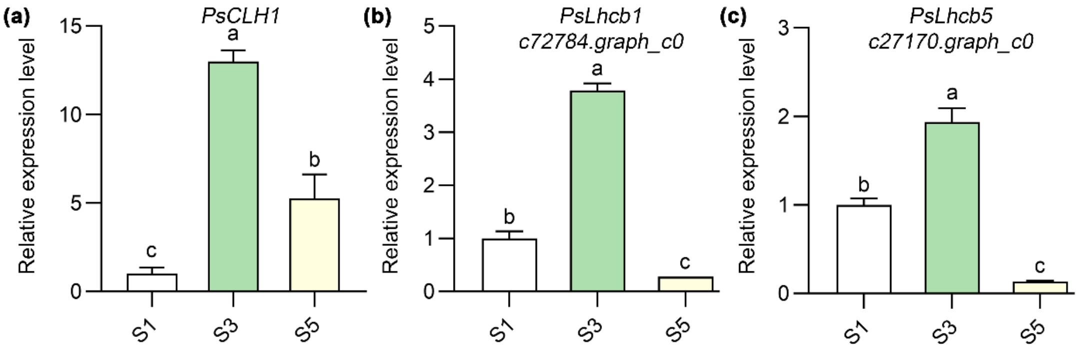

Fig. S4 qRT-PCR analysis of *PsCLH1*, *PsLhcb1*, and *PsLhcb5* at three stages in *P. suffruticosa* 'LMYY' petals. The mean values from three biological replicates are shown. Different letters above each bar denote significant differences according to one-way ANOVA with Tukey's multiple comparisons test ($P < 0.05$).

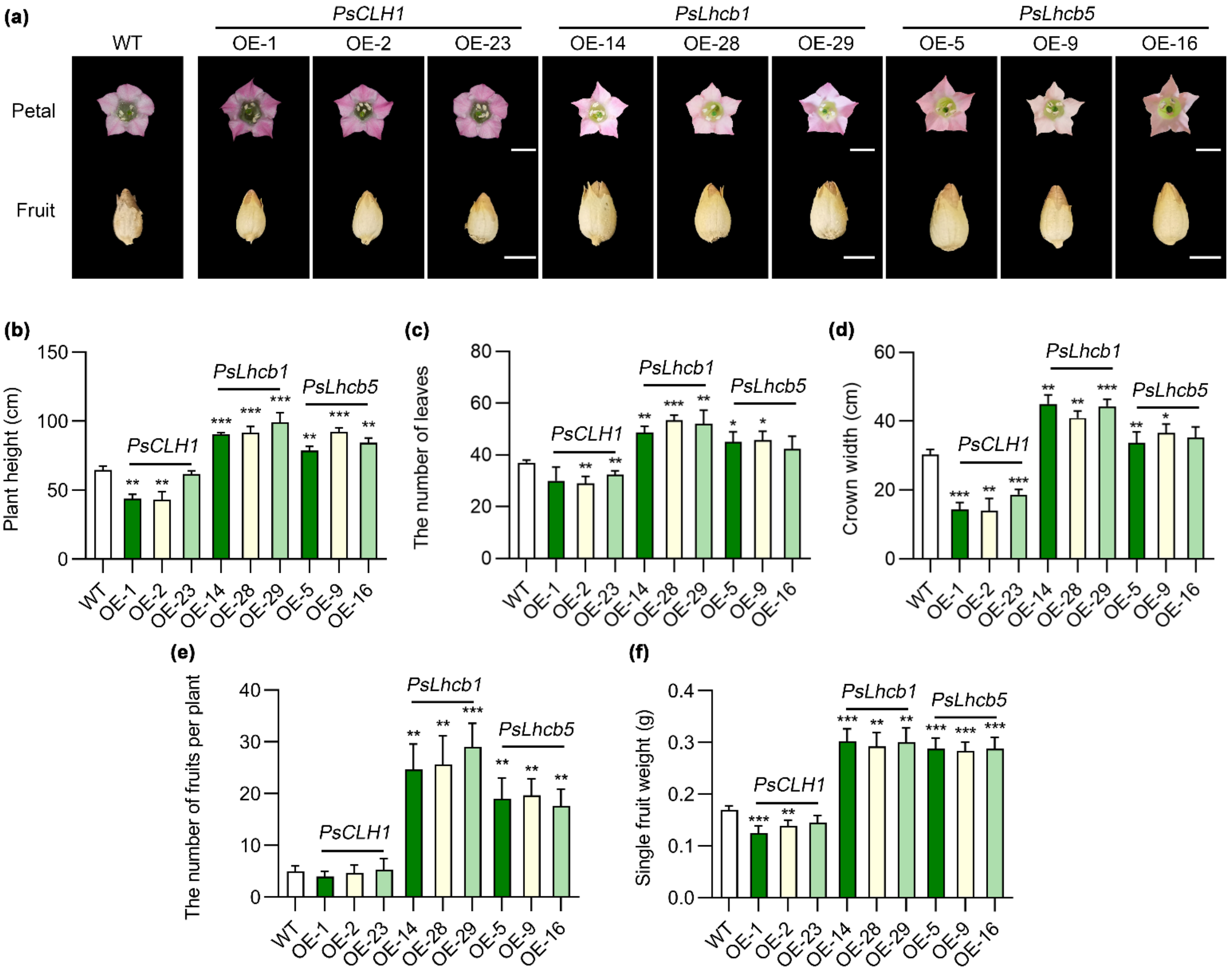

Fig. S5 Overexpression of *PsCLH1*, *PsLhcb1*, and *PsLhcb5* affected tobacco growth and yield. Values are presented as the means ± SD from three biological replicates. Asterisks indicate significantly different values, which are compared with that of WT control (*, $P < 0.05$, **, $P < 0.01$, and ***, $P < 0.001$, two-tailed student's *t*-test). Scale bars = 1 cm.
